# Metastatic colorectal carcinoma mimicking primary ovarian carcinoma presenting as ‘giant’ ovarian tumors in an individual with probable Lynch syndrome: a case report

**DOI:** 10.1186/1752-1947-7-158

**Published:** 2013-06-20

**Authors:** Peter A Ongom, Michael Odida, Robert L Lukande, Josephat Jombwe, Emmanuel Elobu

**Affiliations:** 1 Colorectal Surgery Unit, Department of Surgery, School of Medicine, Makerere College of Health Sciences, Makerere University, P O Box 7072, Kampala, Uganda; 2 Histopathology Unit, Department of Pathology, School of Biomedical Sciences, Makerere College of Health Sciences, Makerere University, P O Box 7072, Kampala, Uganda; 3 Colorectal Surgery Unit, Department of Surgery, Mulago Hospital, P O Box 7051, Kampala, Uganda

**Keywords:** Colorectal cancer, Hereditary non-polyposis colorectal cancer (HNPCC), Lynch syndrome 2, Primary ovarian cancer, Subcutaneous metastases

## Abstract

**Introduction:**

Ovarian metastases occur in 3 to 8% of women with primary colon cancer. In the setting of a pre-existing colorectal carcinoma this would constitute a hereditary non-polyposis colorectal cancer, Lynch 2 syndrome, accounting for 5 to 10% of colon cancer cases. We unveil a case of ‘giant’ ovarian tumors mimicking primary ovarian cancer; ostensibly the first reported in East Africa.

**Case presentation:**

A 58-year-old African woman was diagnosed with colorectal adenocarcinoma in June 2009. She had a right hemicolectomy with the tumor staged as regional cancer, following histopathological examination. Chemotherapy was administered both adjuvantly and 1 year later for what was thought to be a recurrence of tumor. Despite this, her general condition deteriorated. Following re-evaluation and an exploratory laparotomy she was found to have bilateral ‘giant’ ovarian tumors, with peritoneal seedlings and subcutaneous metastases (colonic in origin). A bilateral salpingo-oophorectomy was done, accompanied by histopathological analysis with institution of chemotherapy for ovarian cancer. Following immunohistochemistry tests and microsatellite instability analysis it was found that the ovarian tumors were secondaries from the colon. She was also identified as a Lynch syndrome case or a case of sporadic microsatellite instability, although with no suggestive family cancer history. The treatment regimen was changed to suit metastatic disease.

**Conclusions:**

The case presents a diagnostic and thus treatment conundrum. Two primary tumors (suspected Lynch syndrome) had been perceived yet there is actually only metastatic colorectal cancer. We also have a rare and unusual metastatic presentation: ‘giant’ bilateral ovarian tumors and subcutaneous nodules, concurrently. Further still, she is a case of probable Lynch syndrome, requiring genetic analysis for definitive classification and surveillance for hereditary non-polyposis colorectal cancer-associated cancers.

Important inferences are drawn. Firstly, ‘giant’ ovarian tumors diagnosed as primary ovarian cancer may actually be colonic secondaries. Secondly, immunohistochemistry and microsatellite instability analysis tests ought to be part of the diagnostic package in colon cancer management, particularly for identifying tumor origin and the Lynch syndrome (a condition which has had little attention in resource-limited countries). Thirdly, multidisciplinary team collaboration is emphasized in colorectal cancer management.

## Introduction

Colorectal cancer is among the top five leading forms of cancer among women in Uganda [1]. In some resource-rich countries it is the second leading cause of cancer-related deaths [2]. The majority of colorectal cancer cases are sporadic, occurring in individuals without any known familial predisposition. Approximately 20% of all cases occur with a related family history, but most of the predisposing genetic factors are not yet identified.

The ovaries are an uncommon secondary site for metastatic colorectal carcinoma; the liver and lungs being commoner. In general, the ovaries are the organ of the female reproductive system most commonly affected by metastases [3]. Ovarian metastases occur in 3 to 8% of women with primary colon cancer [4], with their prevalence at the time of diagnosis being 1.1%. Following surgery, the metachronous prevalence remains 1.1% [5]. Colorectal cancer accounts for 65% of the malignancies found to have ovarian metastases at the time of primary surgery. Metastatic gastrointestinal cancers frequently mimic an ovarian primary in both pre- and postmenopausal women [6], yet covert gastrointestinal tumors may present as advanced ovarian cancer. Subcutaneous or skin metastases account for only 5% of metastases [7,8].

The occurrence of a primary ovarian carcinoma, metachronously, with colorectal carcinoma is a well-known entity. It is a presentation of hereditary non-polyposis colorectal cancer (HNPCC; Lynch syndrome), the commonest hereditary type of colon cancer [2], and constitutes 5 to 10% of colorectal cancer cases [9]. There are two phenotypic types of Lynch syndrome: type 1 and 2. Lynch syndrome 1 presents with two separate colorectal cancers, whereas Lynch syndrome 2 manifests as colorectal cancer with another extra-colonic cancer. The extra-colonic cancers are: endometrial, gastric, urinary tract, biliary tract, pancreatic, small bowel, brain and skin. Rarely, ovarian cancer may manifest in Peutz–Jeghers syndrome. Ovarian cancer is the second most common extra-colonic site in women, its overall lifetime risk of development being 6.7% [10].

Lynch syndrome is inherited in an autosomal dominant pattern. Because of the absence of an overt polyposis phenotype, it can be the most challenging hereditary colorectal syndrome to recognize. Germline mutations in one of several deoxyribonucleic acid (DNA) mismatch repair (MMR) genes are responsible for this syndrome. The MMR genes in the presence of microsatellites, define the Lynch syndrome genotype. Recent studies have suggested a median age of diagnosis of associated colorectal cancers of 61.2 years and a lifetime colorectal cancer risk of 52.2% in women [10]. Synchronous and metachronous tumors are frequently observed.

To establish a diagnosis of Lynch syndrome, every patient with colorectal cancer should undergo a detailed family history. A suggestive family history is delineated by the Amsterdam II criteria, and Bethesda guidelines to maximize sensitivity [2]. Specific features that should raise suspicion are: multiple family members affected with colorectal cancer or associated extra-colonic tumors; young age at diagnosis of colon cancer; or multiple Lynch syndrome-associated cancers in a single individual. Currently, fulfillment of the Amsterdam II criteria is insufficient to establish a definitive diagnosis of Lynch syndrome, and molecular testing is required for this purpose. We are very limited in capacity to conduct these tests in Uganda.

## Case presentation

Our patient is a 58-year-old African-Ugandan woman of Bantu ethnicity. She was diagnosed with adenocarcinoma of the colon in June 2009, following her presentation to hospital with features of acute intestinal obstruction. At the time, she had complaints of on-and-off partial constipation for 2 weeks, abdominal pain and distension for 2 days, and vomiting for 1 day. She was dehydrated and wasted. Following resuscitation, an exploratory laparotomy was done at which she was found to have a circumferential tumor of the ascending colon causing obstruction. There was grossly distended bowel proximal to it and mesenteric lymphadenopathy. There was no ascites, and all other abdominopelvic organs were normal. A cecostomy with excisional biopsy of a mesenteric lymph node was done. Histopathologic examination showed an adenocarcinoma within the lymph node.

She had previously been unwell for about 3 years prior to this condition, with on-and-off periods of constipation and loose stool motions, alternately. These episodes lasted for between 1 week and 1 month, with relative periods of wellbeing lasting up to a month. Concurrently, she had periods of abdominal pain. This was diffuse, although largely right-sided, was of insidious onset and dull in nature. There was some pain relief with oral analgesics. Constipation was relieved with a warm soap enema on one occasion. She had never felt any mass per abdomen. There was neither a history of melena stools nor blood in her stool. Despite her good appetite, she had lost weight over a period of about 4 months.

Six weeks after her emergency operation she underwent a definitive standard open right hemicolectomy. She had no ascites, peritoneal seeding or para-aortic lymphadenopathy. Her liver, ovaries, uterus and pouch of Douglas were normal. Histopathologic examination staged the tumor as pT2, N2, M0: Dukes C. She had a relatively quiescent clinical period after approximately 2 months of recovery from the direct effects of surgery. Adjuvant chemotherapy was instituted. She had the Mayo regimen (5-fluorouracil (5-FU) and leucovorin) instituted 3 months after her first presentation, for a period of 6 months. In addition, six cycles of the FOLFOX regimen (5-FU, oxaliplatin and leucovorin) were administered, after the Mayo regimen, for another 6 months. Following this she remained weak and quite limited in carrying out her routine activities. She is a widow with eight children, all alive and well. There is no family history suggestive of colorectal, breast, ovarian, uterine, renal, stomach or skin cancer.

At the end of 2010 she developed gradual abdominal distension, with the feeling of a mass in the hypogastric and left iliac region. This prompted her to return to the oncology unit 4 months later. She described her condition as having generally deteriorated. She felt weaker, spent more time in bed, and reported progressive anorexia and weight loss. Bowel habits were essentially normal. On examination, she was sick looking, wasted and had bilateral pedal edema. There was no significant lymphadenopathy, jaundice or digital clubbing. Abdominal examination revealed asymmetrical distension, normal movement with respiration and the presence of a midline incision scar. She had a right iliolumbar incisional hernia and moderate ascites. There was a mass in the left lower abdominal quadrant, measuring about 15 by 15cm in dimension. It was smooth, regular, firm, mobile and felt to arise from the pelvis to just above the umbilicus. Other systemic examination was unremarkable. A diagnosis of recurrent colorectal carcinoma was made.

An abdominal ultrasound scan and the routine hepatic, renal and hematological tests were done. On ultrasound scan her liver was found to be normal although she had ascites and a right pleural effusion. The other laboratory tests were normal. She was started on capecitabine (oral 5-FU) and leucovorin for 6 months, followed by the FOLFOX regimen again. Throughout this period there was no remarkable improvement. Abdominal distension progressively increased and she had to have regular peritoneocentesis for ascitic relief.

**Figure 1:**
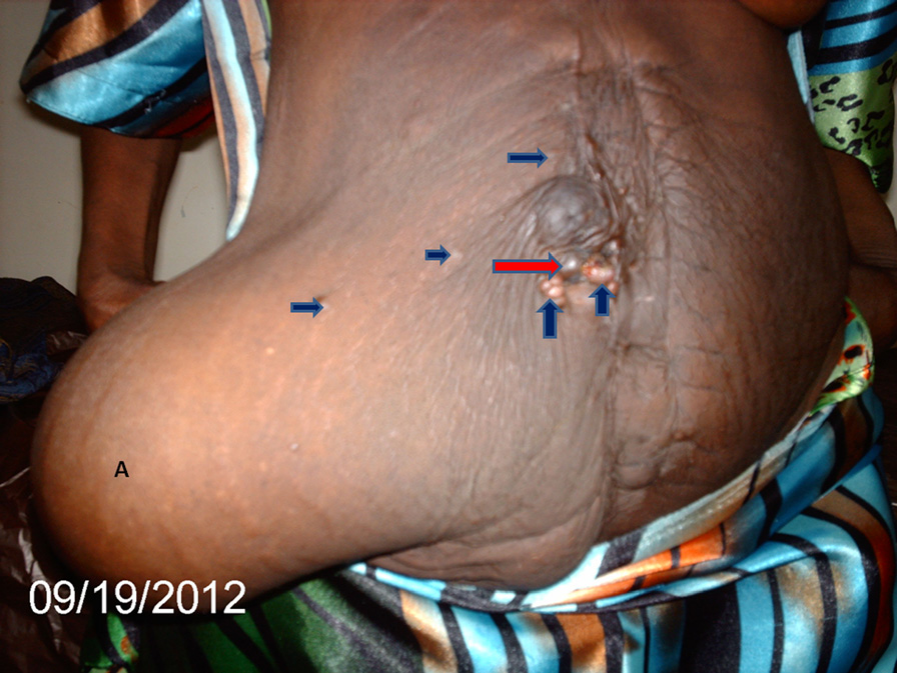
**Photograph after bilateral oophorectomy.** Area marked **A** shows an abdominal wall hernia at site of previous abdominal stoma. It is filled with ascitic fluid. Deep blue arrows point to subcutaneous metastases, whereas red arrow points to umbilical metastasis (Sister Mary Joseph’s nodule).

**Figure 2:**
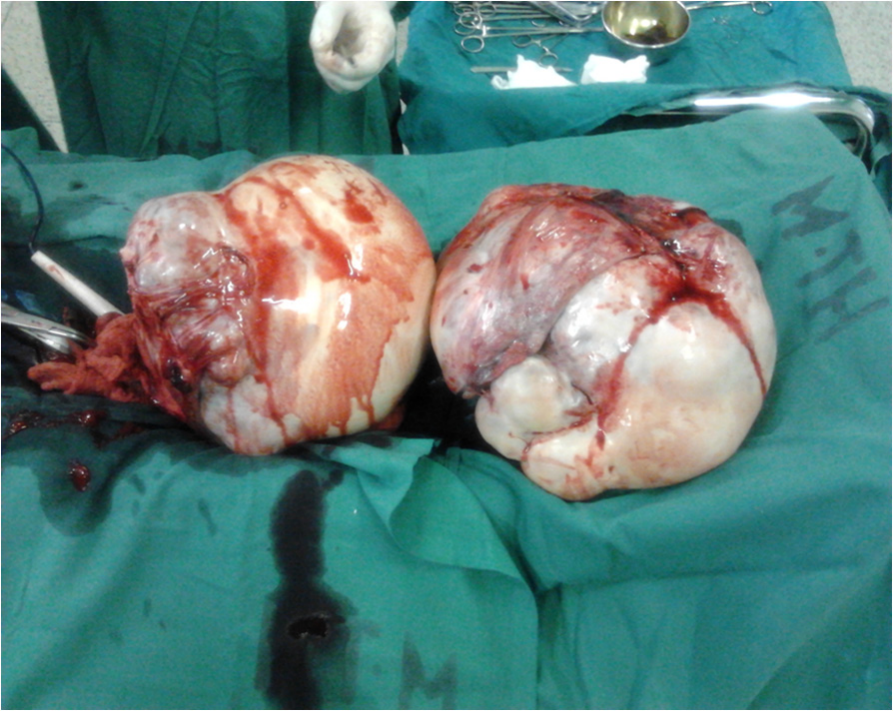
**Photograph of ‘giant’ ovaries following oophorectomy.** Note the relative size of the ovaries compared with neighboring surgical instruments: artery forceps and diathermy pencil.

**Figure 3:**
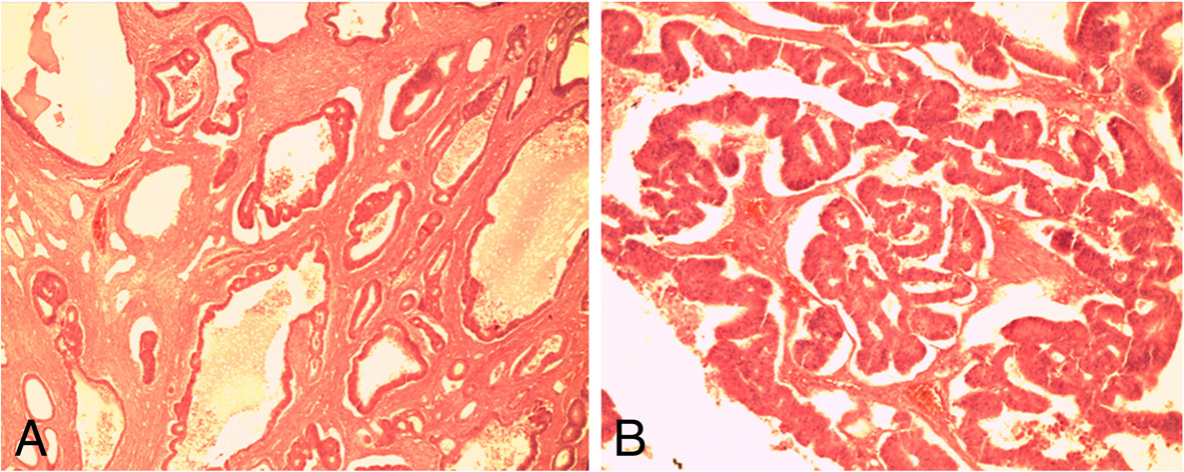
**Hematoxylin and eosin-stained sections of the ovary.** Photomicrograph **A** (under ×4 magnification objective) shows malignant glands in the ovarian stroma. Photomicrograph **B** shows the malignant glands under higher magnification (×10 magnification).

In May 2012 her condition was re-evaluated. She was frail and had grade 2 edema. There was a Sister Mary Joseph’s nodule and scattered periumbilical, hypogastric and epigastric subcutaneous nodules (Figure 1). She had a prominent right-sided incisional hernia in the iliolumbar region. This was the area she had had a cecostomy placed. She had gross ascites and a large mass per abdomen, extending from the pelvis to just about 5cm inferior to the xiphisternum in the epigastrium; most of the abdomen was occupied by it. It was irregular, firm and mobile in the transverse plane. A computerized tomography scan was done and it indicated that the mass (dimensions: 168 × 279 × 273mm) was arising from the pelvis; uterine, ovarian or rectal in origin. Three months later, an exploratory laparotomy was done which revealed bilateral ‘giant’ ovarian tumors (Figure 2). They were freely mobile with normal fallopian tubes, and no attachment to other intra-abdominal or pelvic organs. The uterus was atrophic and free of any tumor infiltration. The liver was regular in texture and consistency, and no masses were palpated. Extensive peritoneal seedlings were present. Bilateral salpingo-oophorectomy was done along with drainage of 3L of hemorrhagic ascitic fluid. The left ovary was larger than the right one with widest diameters being 30cm and 28cm, respectively (Figure 2). Histopathologic findings were interpreted as well-differentiated cystadenocarcinoma of the ovaries (serous type) (Figure 3).

**Figure 4:**
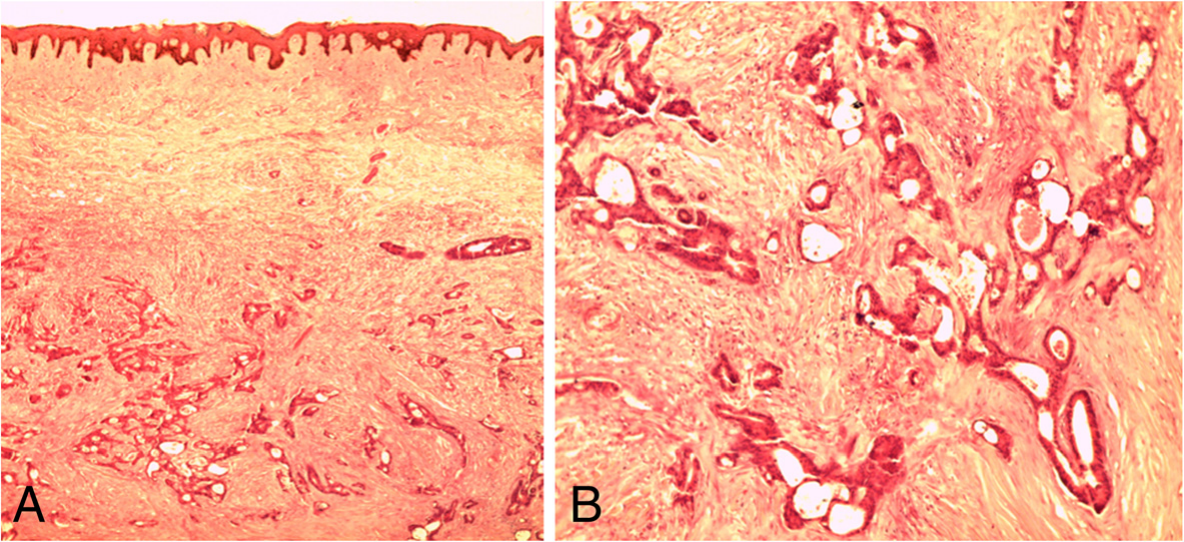
**Hematoxylin and eosin-stained sections of skin.** Photomicrograph **A** (under ×4 magnification objective) shows the reticular dermis being invaded by malignant glands. Photomicrograph **B** shows the malignant glands under higher magnification.

It was thought at the time that this could be a case of either Lynch 2 syndrome (colorectal and ovarian cancer) or metastatic colon cancer. She was then started on a regimen of cisplatin and paclitaxel for the management of ovarian carcinoma. Excision biopsies of her Sister Mary Joseph’s nodule and a subcutaneous epigastric nodule revealed them to be subcutaneous metastases from the colorectal adenocarcinoma (Figure 4). She did not show significant improvement after two cycles of treatment, except for the relief of the mass effect due to the ‘giant’ ovarian tumors. It was decided to revert to treatment for metastatic colon cancer alone (capecitabine). She showed marked improvement and did not have to undergo peritoneocentesis for ascites.

**Figure 5:**
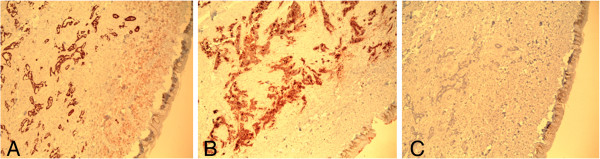
**Immunohistochemically (diaminobenzidine) stained sections of skin (under ×4 magnification).** Photomicrograph **A** shows malignant glands in the dermis staining positively with CDX2. Photomicrograph **B** shows the malignant glands positively stained by carcinoembryonic antigen. Photomicrograph **C** shows negative staining by cancer antigen 125.

**Figure 6:**
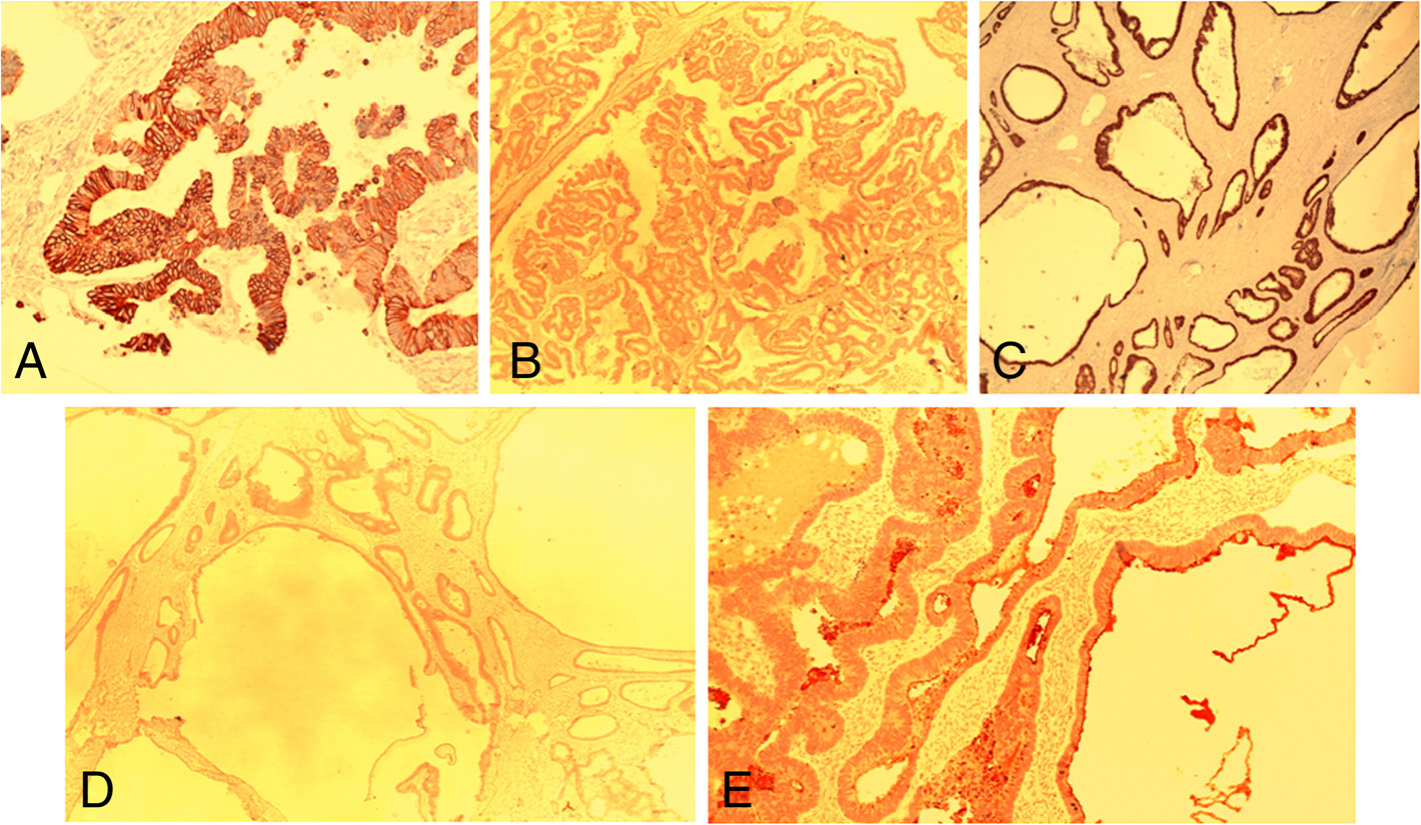
**Immunohistochemically (diaminobenzidine) stained sections of the ovary.** Photomicrograph **A** shows the malignant glands staining positively with cytokeratin (CK) 20 (×20 magnification). Photomicrograph **B** shows the malignant glands negatively stained by cancer antigen 125 (×4 magnification). Photomicrograph **C** shows positive staining with CDX2 (×4 magnification). Photomicrograph **D** shows negative staining with CK 7 (×4 magnification). Photomicrograph **E** shows positive staining with carcinoembryonic antigen (×10 magnification).

In December 2012 we conducted an array of immunohistochemistry (IHC) tests on the excised ovaries and cutaneous lesions (Table 1). Doubts still existed over the origin of the ovarian carcinoma. The cancer antigen (CA)-125 stained negative (Figures 5 and 6). This is typical of primary ovarian mucinous carcinomas and colorectal adenocarcinomas. It stains positive in primary ovarian serous carcinomas. CDX2, a sensitive marker and quite specific for colorectal carcinoma [11], was positive (Figures 5 and 6). Carcinoembryonic antigen (CEA) was also strongly and diffusely positive (Figures 5 and 6), as is usually the case in colorectal carcinomas. The use of the CA-125 to CEA ratio may help to discriminate gastrointestinal carcinoma from ovarian carcinoma [6]. Further IHC with cytokeratin 7 and 20 was negative and positive, respectively (Figure 6), making metastatic colorectal carcinoma the definitive diagnosis [12].

**Table 1:** Immunohistochemistry (diaminobenzidine) staining results

**Immunostain**	**Organ**	
	**Ovary**	**Skin**
**Cancer antigen 125**	Negative	Negative
**CDX2**	Positive	Positive
**Carcinoembryonic antigen**	Positive	Positive
**Cytokeratin 7**	Negative	Not available
**Cytokeratin 20**	Positive	Not available

In addition, we analyzed for microsatellite instability (MSI) using the recommended microsatellite markers. Analysis of MMR genes *MLH1* and *PMS1* was negative, *MSH2* weakly positive, and *MSH6* was strongly positive. This means she could be a case of Lynch syndrome or a sporadic MSI-high (MSI-H) [2]. MSI-H means that two or more markers are positive and at least 30% of the markers show instability. Moreover, the modified Bethesda criteria also consider MSI-H positivity as marking the presence of a MMR gene mutation.

## Discussion

Our patient presents a scenario of multiple tumors, one of which may not be diagnosed with certainty through basic histopathological examination. Colorectal adenocarcinoma was first diagnosed 3 years ago. Following surgery last year, a diagnosis of ovarian carcinoma with cutaneous metastases of the colon carcinoma was made. The initial diagnosis of a primary serous carcinoma of the ovary was only changed to that of colorectal carcinoma with metastases to the ovaries 1 month ago in response to results from IHC tests.

Colorectal carcinoma is most commonly a single primary tumor, with or without metastases. In our patient there are transperitoneal metastases with multiple peritoneal seedlings and cutaneous nodules. Cutaneous metastasis from a visceral malignancy is rare with an incidence of 5.3% [6]. Our patient had a Sister Mary Joseph’s nodule and other nodules around the previous incision and periumbilical area. Previous literature describes this occurrence mostly around the incision scars [8] and it is hypothesized that the mechanism is implantation at the time of surgery [8,13]. Sister Mary Joseph’s nodules are a result of transperitoneal-lymphatic spread. Nodules are the most frequent clinical presentation of cutaneous metastases [13,14].

Going by our working diagnosis of metastatic colorectal cancer and primary ovarian cancer, she was classified as a case of Lynch syndrome 2. Women with HNPCC-associated colorectal cancer have a 12% lifetime risk for ovarian cancer, meaning several of them will have more than one cancer in their lifetime [15]. The only tumor commoner than ovarian cancer, is endometrial cancer. Confirmation of HNPCC is done with molecular tumor tests and blood genetic testing. Although we neither conducted comprehensive molecular tests for MMR proteins nor blood genetic tests, we could still be dealing with a Lynch syndrome given our other test results. However, it could still be a sporadic case because up to 15% of them are also associated with MSI. Further still we have no suggestive family history. Of importance though, family disease history and records are highly unreliable in the East African region.

If indeed it is a case of Lynch syndrome (confirmed through MMR protein and blood genetic tests), this whole scenario may mean she has not yet developed a second tumor (metachronous), or may already have one; still covert. A screening strategy for common HNPCC-associated cancers can then be instituted.

The conundrum of this case is the course of management in relation to the unfolding clinical features following the surgical treatment for the colorectal cancer (extended right hemicolectomy) when put in context of the histopathological diagnosis. Adjuvant chemotherapy would be expected to deal well with the colorectal carcinoma. Development of new abdominal symptoms after adjuvant treatment pointed to a possible recurrence. Failure to improve after more chemotherapy could point to a ‘resistant’ form of tumor, an inappropriate regimen and/or a wrong initial diagnosis. A reassessment was called for and done, resulting in another laparotomy with a clinical diagnosis of ‘giant’ ovarian tumors (Figure 2). Subsequent histopathological examination showed a second independent tumor: primary ovarian carcinoma. Up to 45% of secondary ovarian tumors are clinically seen to be primary ovarian carcinomas and many are misinterpreted as such on pathologic examination even when there is a known intestinal carcinoma [16]. Failure to improve on the cisplatin regimen led to reversion to capecitabine. The benefits of this decision, coupled with the salpingo-oophorectomy, are evident in the patient’s clinical recovery. The surgery alone provided massive cytoreduction of tumor. Bilateral oophorectomy for ovarian metastasis from colorectal cancer has a good impact in providing a disease-free period and improving overall survival [4]. The current capecitabine regimen is dealing quite well with the other peritoneal metastases. However, we still treat this disease within the context of a poor prognosis. Peritoneal dissemination is an adverse prognostic factor [17].

While the patient continues treatment for metastatic colorectal cancer, the possibility that we are dealing with a Lynch 2 syndrome remains at the front of our minds. There is a need to confirm the condition with blood genetic analysis and/or continue with surveillance for other tumors. In the event that Lynch syndrome is confirmed, our first step is counseling of the patient and her relatives. Prophylactic hysterectomy is an option if she is fit for surgery. Surveillance for other tumors includes the following: colonoscopy of the remaining colon annually; gastric and duodenal cancer screening – upper gastrointestinal endoscopy annually; urothelial cancer screening – annual urinalysis (cytology); and central nervous system cancer screening – annual physical examination [2].

## Conclusions

Colorectal cancer in Uganda and the East African region is getting increasingly recognized. More cases are being diagnosed and treated by multidisciplinary teams of surgeons, medical oncologists, radiotherapists and pathologists. Adequate documentation of patterns of colorectal cancer and HNPCC-associated cancers is lacking. There are no records of family trees of what are now described as HNPCC families. Our case highlights several important issues encountered in the management of colorectal cancer. This patient alone has shown the following: firstly, a probable Lynch syndrome (at least the presence of MMR gene mutation is evident) or a sporadic MSI-H case; secondly, tumors can mimic others; and lastly, cutaneous and bilateral ‘giant’ ovarian metastases from colorectal cancer can arise contemporaneously. This is an unexpected and unusual metastatic presentation.

The following learning points can be gathered from this case: IHC and MSI analysis tests ought to be part of the diagnostic package in management of colorectal cancer; multidisciplinary team collaboration is of paramount importance; and, other specialists not often involved, may frequently become core members of the team (gynecologists, urologists, dermatologists and neurosurgeons) both in treatment and surveillance. In our setting, the role of IHC in distinguishing ovarian metastases from primary ovarian tumors, and the occurrence of MMR deficiencies needs emphasis.

## Consent

Written informed consent was obtained from the patient for publication of this case report and all accompanying images. A copy of the written consent is available for review by the Editor-in-Chief of this journal.

## Competing interests

The authors declare that they have no competing interests.

## Authors’ contributions

PAO conceptualized the idea, wrote the manuscript, managed our patient peri-operatively, and performed operations. RLL co-wrote and edited the manuscript, and conducted the histopathological analysis. MO conducted histopathological analysis and edited the manuscript. JJ and EE operated on and managed the patient. All authors read and approved the final manuscript.
